# Last Aid Courses as a Means for Public Palliative Care Education—A Narrative Review of the Literature and 10 Years of Experience Around the World with Implications for Future Research

**DOI:** 10.3390/healthcare14010096

**Published:** 2025-12-31

**Authors:** Georg Bollig, Jason Mills, Sindy Müller-Koch, Pandeli Pani, Bianca Neumann, Erika Zelko

**Affiliations:** 1 Department of Anesthesiology, Intensive Care, Palliative Medicine and Pain Therapy, Helios Klinikum Schleswig, 24837 Schleswig, Germany; 2 Department of Palliative Medicine, Faculty of Medicine and University Hospital, University of Cologne, 50937 Cologne, Germany; 3 Last Aid Research Group International (LARGI), 24837 Schleswig, Germany; 4 Letzte Hilfe Deutschland gGmbH, 24837 Schleswig, Germany; 5 Research Centre for Palliative Care, Death, and Dying (RePaDD), College of Nursing and Health Science, Flinders University, Adelaide 5042, Australia; 6 Nursing Home am Lerchenberg, 06886 Wittenberg, Germany; 7 Deutsche Welle, 53113 Bonn, Germany; 8 Sue Ryder, London NW1 1BU, UK; 9 International Observatory on End of Life Care, Division of Health Research, Faculty of Health and Medicine, Health Innovation One, Sir John Fisher Drive, Lancaster University, Lancaster LA1 4AT, UK; 10 Institute for Palliative Medicine, Medical Faculty, University Maribor, 2000 Maribor, Slovenia; 11 Institute for General Medicine, Medical Faculty, Johannes Keppler University, 4040 Linz, Austria

**Keywords:** palliative care, public palliative care education, end-of-life care, compassionate communities, Last Aid Course, health literacy, death literacy, grief literacy, education

## Abstract

**Objective**: To provide a narrative overview of the scientific knowledge on Last Aid Courses and experiences from different countries. **Background**: The levels of death literacy, grief literacy, and knowledge about palliative care are low in many countries around the world. For many people, dying, death, and grief are still a taboo. Public Palliative Care Education (PPCE), the public knowledge approach, and the Last Aid Course (LAC) aim to increase death literacy, grief literacy, and public knowledge about palliative care. **Methods**: A literature search in the databases PubMed/Medline, CINAHL, and PsycInfo was undertaken. Other additional sources were found by hand searching, books, reference lists, and the internet. A narrative overview of the existing literature on LAC and Public Palliative Care Education (PPCE) is provided. Experiences with PPCE and LAC from different countries are presented. Based on the findings, a future agenda for research on PPCE and LAC is presented. **Results and Discussion**: PPCE and LAC have been introduced in 23 countries. A total of 17 articles and reviews on Last Aid were included. Research on the effects of LAC in different countries and cultural issues connected to LAC are ongoing. **Conclusions**: Since 2015, LACs have been introduced in 23 different countries. The LAC, the LAC-KT, and PPCE may enhance the public debate on dying, death, grief, and palliative care and may empower people to contribute to end-of-life care in the community. Future research on PPCE, the LAC, and the LAC-KT should focus on retention over time and the long-term effects of the courses.

## 1. Introduction

The global demand for palliative and end-of-life care is expected to rise in the coming decades due to the growing number of chronically ill, elderly, and frail individuals worldwide [1,2,3,4,5,6,7,8]. Moreover, as most people express a preference for spending their final days at home, the need for home-based palliative care will also continue to increase around the world [9,10,11,12]. Due to the limited resources of the health care services in communities, several authors have recommended a public health palliative care approach, including citizens in palliative care provision in the community [13,14,15,16]. Kellehear posed the statement that “end-of-life care is everyone’s business”, meaning that everyone in the community should be engaged in palliative care provision in the community in cooperation with health care professionals [14,15]. A key problem and barrier for palliative care provision by lay persons in the community is the lack of public awareness around palliative care, death literacy, and grief literacy [17,18,19,20,21,22,23,24,25,26,27,28]. The first step for people to engage in palliative care provision in the community is awareness and the acquisition of basic knowledge on palliative care, which can be obtained from Last Aid Courses (LACs) for both lay people and professionals from healthcare and social services, as described by Bollig as the public knowledge approach to palliative care. This approach includes health, death, and grief literacy as parts of Public Palliative Care Education (PPCE) for everyone that ideally should already start in childhood [13,29].

Across many countries, broader social and demographic shifts are influencing how people understand and engage with death, dying, and care at the end of life. Many countries are also grappling with the realities of an ageing population, where growing numbers of older people are living with long-term conditions while health and social care services face increasing pressure [30]. At the same time, it has been shown how everyday caring roles have gradually moved from families and neighbourhoods into professional services, leaving many people feeling uncertain about how to help when someone is dying [31,32]. These changes sit alongside persistent gaps in public preparedness for dying and bereavement, with population studies reporting low levels of death literacy and limited awareness of palliative care or advance planning [33]. Research also suggests that people who have direct experience of caregiving or community involvement often develop a stronger sense of confidence and readiness around end-of-life issues, pointing to the value of experiential learning [34,35]. Hospices have increasingly responded to these needs by expanding their educational role. Hospices today are not only training health and social-care professionals but also offering outreach to schools, community groups, and local organisations to strengthen public confidence, communication, and practical skills around serious illness and bereavement. While such hospice-led programmes reflect well-established local approaches, recent international evidence highlights that low public and professional literacy about end-of-life care remains a major global barrier to equitable palliative care access, and that basic education for the wider health and social-care workforce is an urgent priority [8]. Due to a lack of awareness for palliative care and low death and grief literacy in the public, a number of experts have recommended education of the public [13,17,18,19,20,21,22]. Different experts and the Lancet Commission on the Value of Death have recommended improved death literacy, knowledge, and skills in end-of-life care for everyone [13,17,28,29]. In 1986, the World Health Organization’s Ottawa Charter for Health Promotion recommended that the collaboration between health care services and communities should be an essential part of public health, including end-of-life care [36]. The long-established community-owned palliative care model in Kerala [37] demonstrates that when education and basic training are widely available, community-based approaches to end-of-life care can be implemented at scale, with volunteers, families, and local organisations taking on meaningful roles in awareness-raising, practical support and care. In several European countries, nationally coordinated Public Palliative Care Education initiatives, such as Last Aid, have been developed to address similar challenges by providing accessible, standardised education for the general population. (Inter)national programmes like these can complement and strengthen local efforts by offering consistent, population-level learning opportunities that help bridge the gaps created by uneven resources, geographical variation, and differing levels of community engagement. Because these programmes are led by international steering groups that review and update course content on a regular basis, they can also help reduce bias and guard against knowledge inertia, ensuring that public education remains consistent with current evidence and diverse cultural perspectives. These wider insights offer an important foundation for understanding why Public Palliative Care Education has an increasingly significant role in strengthening community capacity and supporting shared involvement in end-of-life care. These international programmes can also help sustain public engagement and learning in settings where local resources are limited, offering a level of continuity that can be difficult for individual hospices or community organisations to maintain. When funding, staffing, or organisational capacity fluctuate, it is often the more resource-intensive activities, such as research, ongoing curriculum development, and publication, that are hardest to continue. Broader international initiatives can act as a stable framework that complements and supports local provision during these periods of strain.

Last Aid Courses (LACs), Last Aid Courses for kids and teens (LAC-KTs), and the public knowledge approach suggested by Bollig aim to enhance the public discussion on serious illness, dying, death, and grief and to provide the public with basic knowledge and skills in palliative care [13,29]. Last Aid Courses can be seen as building the knowledge base for compassionate communities to enable and empower the public for palliative care provision in the community [13,29]. LACs are standardized courses with four teaching modules with 45 min each that are taught by two certified Last Aid Course instructors with experience in the field of palliative care. The LAC is usually provided in a classroom setting with six to twenty participants but can also be held as an online course. It includes presentations, discussions, and practical training. An international Last Aid working group is responsible for a consensus on the standardized course curriculum and the course contents. National differences that need to be addressed and included in the national slide presentation are topic legislation, advance care planning, and the national organisation of palliative care provision [13]. Table 1 provides an overview of the four modules of the LAC and their contents.

Since the first introduction of the Last Aid Courses in Norway, Denmark, and Germany in 2014/2015, standardized LACs have been started in 23 countries in Europe, Canada, Brazil, Australia, and Singapore. In 2019, the Last Aid Research Group International (LARGI) was founded during the First International Last Aid Conference in Sønderborg in Denmark. The LARGI is a cooperation of international researchers and works on research on the views of participants and LAC instructors as well as the effects of the LAC worldwide. The German NGO Letzte Hilfe Deutschland gGmbH celebrated the 10th anniversary of Last Aid Courses for the public in Germany with a symposium in Schleswig in May 2025. After a decade of implementing the public knowledge approach for palliative care and LACs across multiple countries, it is appropriate to reflect on and synthesize the accumulated experience.

Aim of the study: To provide a narrative overview of the scientific knowledge on Last Aid Courses and the experiences from different countries using Public Palliative Care Education (PPCE) as framework.

## 2. Materials and Methods

The current narrative review builds on a review that was previously published four years ago in 2021 [13]. To update and advance knowledge with a more precise context, the focus of this narrative review is on scientific publications about Last Aid and Public Palliative Care Education published between January 2015 and September 2025. First, a systematic search of the peer-reviewed literature was conducted using a predefined search strategy. PubMed, Medline, CINAHL, and PsycInfo databases were searched using the keyword “last aid” and MeSH (Medical Subject Heading) terms “palliative care education” and “public”. All searches were limited to full-text articles published between 2015 and September 2025 in English language peer-reviewed journals. In addition to this, other sources were found by searching the internet, hand searching, books, and reference lists of papers and book chapters on the above-mentioned topics. Sources that were searched for this narrative review included the following:

- Searches in the databases PubMed and Medline, CINAHL, and PsycInfo;
- Internet searches;
- Hand searches from reference lists of retrieved literature;
- Written and oral reports of personal experiences from members of the International Last Aid working group, international experts, and the first author from the implementation of Public Palliative Care Education using Last Aid Courses in different countries.

Selection criteria for inclusion in the review were as follows: only articles in English published in scientific journals that fulfilled the inclusion criteria were included. The inclusion criteria used were as follows: publications focused on Last Aid Courses or Public Palliative Care Education for citizens. All other publications were excluded. Screening was undertaken by multiple reviewers to ensure that only those papers meeting inclusion criteria were included, and agreement was reached by discussion if differences were identified. Assessment of the presented articles included type of publication (original article, review, etc.), main topic of the paper, evaluation method used (quantitative, qualitative, etc.), informants and response rate, main findings, and involved countries. The included articles are presented in Table 2 based on the above-mentioned criteria. An overview of the findings from the literature search and experiences from the implementation process will be presented under thematically arranged subthemes in the results. The methods used for data collection, analysis, and reporting follow the recommendations for narrative literature reviews proposed by Green, Johnson, and Adams [38]. In addition, the narrative review checklist was used for reporting the findings from the literature search [38]. Public Health Palliative Care (PHPC) and Public Palliative Care Education (PPCE) were used as a guiding framework to inform the review. PPCE is an approach to teach the public about dying, death, grief, and palliative care [39] and combines different literacies including health, death, and grief literacy. Through this combined approach, societies shall be empowered to talk openly about these topics and encouraged to participate in the care of people in need of palliative care in the community.

## 3. Results and Discussion

The search strategy retrieved numerous publications on the topics of Last Aid Courses and/or Public Palliative Care Education. The first step of the assessment of the search results was the removal of duplicates. In addition to the literature search in scientific databases, a list of Last Aid publications from the homepage of Last Aid Germany [55], updated 27 September 2025 and including 65 publications on Last Aid (47 articles in journal, 13 books or book chapters and 5 other publications), was assessed and led to the inclusion of 12 articles reporting scientific studies and 5 reviews in Table 2 as well as other publications in journals and book chapters in the current review. Of the 65 listed publications, 62 were published in 2015 or later. This shows that there is a large interest in Last Aid Courses, resulting in a large number of publications on Last Aid Courses that have been published within the last 10 years, after the first Last Aid Courses were started in Norway in November 2014 and Germany in January 2015.

The literature search identified several articles on Last Aid studies and reviews. Table 2 provides an overview of 17 Last Aid studies and reviews published in English in scientific journals [13,29,40,41,42,43,44,45,46,47,48,49,50,51,52,53,54]. The presented articles are ordered by publication year with scientific articles first (*n* = 12) followed by reviews (*n* = 5).

The main results from the included studies were that LACs, LAC-KTs, and online Last Aid Courses (OLACs) are feasible and well accepted by different populations from different countries. Many participants feel encouraged to talk more openly about death and dying and to engage in end-of-life care provision in their community in the future. The first multicentre study on the LAC published in 2021 showed that 88% of the participants were female with a median age of 56 years [29]. Most participants (76%) rated the course as very good and 99% would recommend the course to others [29]. These findings are similar to the results of a scoping review on different death education programs that concluded that death education leads to “a higher willingness to discuss end-of-life decisions and decreased death anxiety, death avoidance, and fear of death” [56]. Interestingly, 9.4% of the 5469 participants of the above-mentioned study [29] had a medical profession, indicating an interest in talking about dying, death, and grief although belonging to a medical profession that may implicate a closer relationship to these topics than for the public. The reason for this need could be a lack of these topics during medical training.

A study on an LAC for employees of a German university hospital revealed that a number of members of the medical staff found the LAC more suitable for educating non-medical hospital staff about care for the dying and requested courses with an extended curriculum in order to meet their learning goals [43]. Based on these findings and wishes, a working group in Germany established a one-day Last Aid Course Professional for workers from health and social care. The first results of the pilot study have been published in German and received a prize for the best publication in the German Journal of Palliative Medicine in the year 2023, showing the recognition and impact within the scientific field of palliative medicine [55]. The results showed that the course is feasible, and that many of the participants rated the course as useful for healthcare personnel and appreciated new perspectives, reflections, and the interactive work on different options for action in palliative care.

A study on cultural differences across the German–Danish border showed that the informants found individual differences more important than cultural differences in end-of-life care but they described differences connected to regulations and organization of services across the border [44]. The informants suggested including the topics of organization and support across the border, religions, and cultures, and especially, advice on how to support people in grief [44]. LACs are welcome and useful in different countries and cultures [29,42,43,44,45,46,48].

A qualitative pilot study of caring relatives suggests that the LAC is seen as a safe place for discussion, empowers caring relatives, and strengthens their skills [47]. The LAC may also contribute to reclaiming agency [48]. Furthermore, the research on LAC indicates that informal caring for relatives is often provided by women [29,48]. The continuing development, quality assurance, and improvement of the course content through research is an important characteristic of the Last Aid project [50]. In addition to empowering LAC participants to engage in palliative care, the LAC influences all five principles of social space orientation and may thus contribute to improved palliative care in the community in the long term [50]. This impact includes effects on the framework of social space and its five principles of resource orientation, participation, networking, sustainability, and contextuality [50]. LAC can, e.g., lead to improved participation of lay people in end-of-life care in the community and the connection and adaptation of local networks of care [50]. Children and teenagers are also interested in talking about dying, death, and grief and appreciate the LAC-KT as an opportunity to talk and learn more about these topics [40,49]. Most of the participating children and teenagers aged 7 to 17 years (84%) already had experience with death and dying before taking the course and 91% found the LAC-KT helpful for everyone [49]. OLACs were introduced in Germany, Brazil, and Scotland during the COVID-19 pandemic in order to keep up with teaching despite meeting restrictions [41,42,46]. The results showed that although the online format is often only seen as the second-best option, it is possible to implement, meaningful, and even led to an increased participation of people who care for others at home and younger people [42]. According to MacAden et al., “Provision of foundational death literacy education in social contexts enhances the personal knowledge, skills, and confidence of individual community members and supports the notion that this personal growth could lead to strengthened community action.”, and they suggested “to include LAC as the foundational core training to promote death literacy in communities.” [46].

Since the start of the implementation of the first Last Aid Courses in Norway, Germany, and Denmark in 2014/2015, three reviews on LACs have been performed and published by Bollig et al. [13,51,52]. The main findings from these reviews were that the LAC as a simple approach to PPCE is feasible and well accepted in different countries. Both research and practical experience from the implementation indicate that the LAC can assist in improving the public debate on death, dying, and palliative care and may also contribute to the empowerment of citizens in the provision of end-of-life care. Two recent reviews published in 2025 have focused on PPCE for the general public [53,54]. One of them, published by Elder, about education for adolescents and young adults concluded that “a variety of designs, methods, and strategies were all identified to have positive participation, feedback, and experiences from high school AYA being provided palliative care curriculum” and that “the use of subject matter experts within palliative care curriculum for high school students is a core characteristic in meeting the World Health Organizations recommendation to embed palliative care curriculum into public awareness strategies” [53]. Seckin et al. [54] describe six main themes of palliative and end-of-life education for the public. These include foundational concepts and philosophies, communication and decision-making, planning and preparation, symptom management, end-of-life care practices, and caregiving support. Interestingly, only three educational concepts for the public include all six themes, one of them being the LAC [54].

In addition to the papers described in Table 2, other publications connected to Last Aid Courses included reports on international Last Aid conferences [55], editorials, comments, opinions, and letters on PPCE and LAC [55,57,58]. The experiences from different countries with the implementation of the LAC show that the LAC is feasible in different settings and across different cultures and ethnicities [13,29,45,46,48]. It has also been shown that different organisations, e.g., palliative care organisations, universities, or the church, are possible national cooperating organisations for the implementation and organisation of LACs in different countries [55]. It has been stated that cultural beliefs and rituals may help to alleviate grief [59]. These topics are addressed in both the LAC and the LAC-KT and are introduced and discussed with the participants [13]. First Aid training for the public, including adults, elderly people, teenagers, and children, is a useful public health initiative and experts recommend it even as mandatory in schools or workplaces [60,61,62,63,64,65]. Some articles on Last Aid recommend implementing Last Aid Courses in an analogous fashion to First Aid courses in the public sphere [13,29,43,49,50]. Thus, Last Aid Courses are an essential part of Public Health Palliative Care. To raise the public awareness for palliative care is very important as the general public is lacking a detailed understanding of palliative care [66] and education and training have been suggested to raise awareness and to improve participation of the local communities in palliative care provision [67]. Talking about death and dying may support the grieving process of teenagers [68]. These findings are similar to the findings from the LAC-KT for teenagers that show that 84% of them have already had experience with dying and death and they want to talk about death and dying [13,49]. Importantly, however, there are apparent gaps in the literature and evidence base for Public Palliative Care Education, and these should be addressed in line with the recommendations made below.

### 3.1. Need for Future Research

The research on LACs, including different course formats and the effects of the LAC, is part of the international Last Aid project and is coordinated by the Last Aid Research Group International (LARGI) that was founded in Sønderborg, Denmark during the first International Last Aid Conference, 2019. LARGI is led by Erika Zelko and Georg Bollig.

#### 3.1.1. Areas and Topics for Future Research on Last Aid

**Figure 1:**
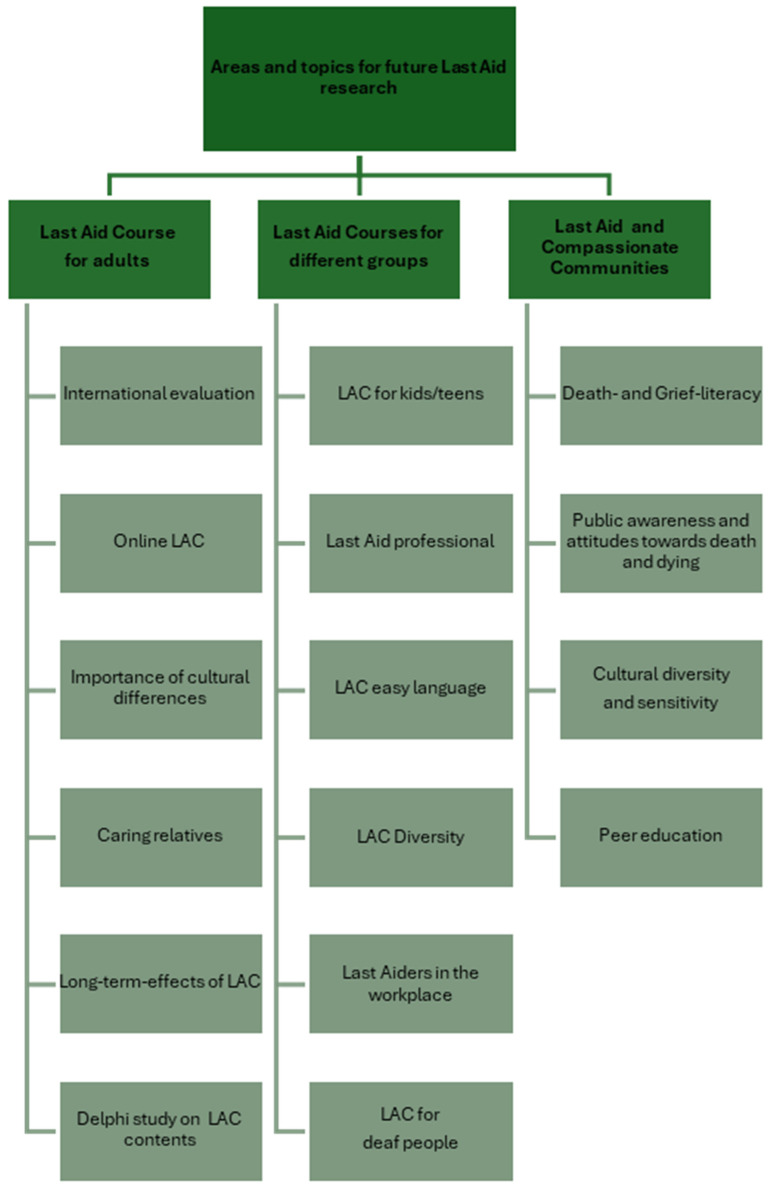
Areas and topics for future Last Aid research.

Based on the findings of the current review and discussions of the experts from the International Last Aid working group and the members of LARGI, Figure 1 presents the topics that are most important for future research on the international Last Aid Course project. Three areas are of outmost importance for Last Aid research in the future: LAC for adults, LAC for different groups, and the interaction between LAC and compassionate communities. In addition to the research on LAC for adults in different countries, the effect on caring relatives and the long-term effects of LAC demand further research—as does the investigation of knowledge retention over time. An international Delphi study on the contents of PPCE and the LAC with the participation of international experts would be useful for the further development of the LAC. As different LAC course formats for different groups have been developed in recent years, these also need a scientific evaluation to support the future improvement and wide implementation of these formats. A prototype of international cooperation and the need for cultural adaptation will be a planned project to translate the LAC-KT into different languages and for its implementation in different countries and across different groups and cultures. In order to improve community-based palliative care, education of the public is needed [21,28,29]. Therefore, the interaction between educational initiatives as the LAC and compassionate communities needs more research. Of special interest, is the evaluation of possible positive effects of the LAC on the death and grief literacy of the public and public awareness of dying, death, grief, and palliative care in general. Other interesting topics are the higher percentage of female LAC participants [29] and thus, future strategies to recruit more men to participate in the LAC. It has been shown that informal carers face many burdens at the end-of-life and that ways to address grief and bereavement and support employees who are informal carers are needed in the workplace [69,70,71]. This is also described for employees who have experienced perinatal loss [72]. The European project EU-CoWork is currently trying to develop compassionate workplace programs to address the above-mentioned topics in the workplace [73]. The need to address dying, death, and grief in the workplace has also been found in a project by the Center for Palliative Medicine at the University of Cologne [74]. The next step of this project shall be the implementation of Lastaiders in the workplace, similarly to Firstaiders. A very interesting question is whether the LAC may contribute to an increased number of people who will die at their preferred place of death, for example, due to an improved possibility of home death that may be enabled through more support from the public after LAC training.

#### 3.1.2. Last Aid as Part of Public Health Palliative Care and Compassionate Communities

An explanatory lens for understanding public reluctance to engage with end-of-life care thoughts and initiatives such as Last Aid is provided by Terror Management Theory [75]. It posits that human awareness of mortality provokes deep existential anxiety, which individuals and societies manage through cultural systems that offer meaning and a sense of symbolic immortality. In contemporary Western contexts, this often takes the form of the medicalisation and professionalisation of death, where dying is displaced into institutional settings and managed by experts, reducing direct and normal confrontation with mortality. This psychological mechanism reinforces a service-seeking culture, in which people defer responsibility for dying and grief to healthcare systems rather than viewing them as shared social experiences. From a critical realist perspective, such mortality-defence processes operate as underlying generative mechanisms that constrain participation in Public Palliative Care Education while simultaneously highlighting its necessity. Last Aid Courses, by normalizing discussion about death and re-embedding care within community life, can be seen as gentle counter-measures to these cultural defences, supporting both personal death awareness and collective capacity to care [13,29,50]. Within the United Kingdom, for example, these psychological and cultural mechanisms intersect with the structural realities outlined in Professor Chris Whitty’s Chief Medical Officer’s Annual Report 2023 [30]. He emphasises that the central public health challenge of ageing is not simply extending lifespan but enhancing independence, quality of life, and community capacity in the later years of life. From a critical realist standpoint, this vision reveals how macro-level structures, including health and social care integration, local infrastructure, and socioeconomic inequalities mediate the possibilities for community engagement at the end of life. While the service-seeking orientation and mortality-defence mechanisms described above may inhibit public participation, Whitty’s framework points to potential enabling conditions for change: strengthening community assets, reducing isolation, and supporting intergenerational engagement. Building on these insights, Last Aid as an option for Public Palliative Care Education can be understood as a practical expression of Public Health Palliative Care (PHPC) principles, translating the values of participation, empowerment, and equity into accessible community education. Yet, the literature to date has focused primarily on feasibility and satisfaction, leaving underexplored the social infrastructure that Last Aid helps to create, encompassing the networks, norms, and shared vocabularies that enable collective care. Equally, questions of equity and access remain largely unexamined—particularly who remains excluded from participation due to socioeconomic, cultural, or geographical barriers. Increasing access through a variety of practical means will be important. For example, the use of novel educational approaches such as immersive simulation, gaming, or escape rooms may assist in improving accessibility and engagement around end-of-life issues. Addressing these gaps will be essential to ensure that Last Aid realises its PHPC potential as an inclusive, community-empowering intervention. In addition to the proposals for future research described above, future research should therefore also move beyond individual-level outcomes, to examine how Last Aid contributes to sustainable community capacity and long-term cultural change. Conceptually, this may be understood as a double-loop process [76], in which the first loop enhances individual health, death, and grief literacy, while the second loop reshapes the social norms and institutional arrangements that govern how dying and bereavement are collectively managed. In this way, Last Aid Courses could act as meso-level mechanisms linking individual reflection with community action, translating policy aspirations for compassionate, age-friendly societies into practice, and exemplifying how PHPC can be lived in everyday life. In conclusion, Last Aid Courses can be seen as building the knowledge base for compassionate communities to enable and empower the public for palliative care provision in the community [13,29].

## 4. Limitations

The most important limitation of this review is the limited number of publication on the LAC and LAC-KT. Most articles on the LAC and LAC-KT have been published by Bollig and/or other authors who are participants of the Last Aid Research Group International (LARGI) that was established during the First. International Last Aid Conference in Sønderborg in Denmark and which is led by Zelko and Bollig. Therefore, this article has a relatively high number of self-citations, which are not avoidable due to the limited number of scientific articles on the topic. The personal experiences of most of the authors can also be seen as a strength of the study as they have both personal experiences with LAC and an in-depth knowledge of the background, practice, and research in the field of PPCE. Further, there is potential for publication bias within the literature reviewed, largely originating from European/Western populations, and findings may not necessarily be generalizable to other geographical contexts and populations. Other possible limitations may be connected to the fact that most LAC participants are female, as shown by Bollig et al. [29], and missing information on the participants’ backgrounds in terms of culture and diversity. Information about the recruiting strategies in different regions and countries is lacking or not reported in most articles. As the strategies used are very different and include school classes and preexisting groups of people as well as LACs that are open for all members of the public, this may be an area of interest for the future. These limitations could be addressed in future research on Last Aid.

## 5. Conclusions

Last Aid Courses have been introduced in 23 different countries since 2015. The LAC, the LAC-KT, and PPCE may enhance the public debate on dying, death, grief, and palliative care and may empower people to contribute to end-of-life care in the community. Future research should explore three main topics: 1. Last Aid Courses for adults including the long-term effects of the courses and a Delphi-study on the contents; 2. LACs for different groups including, e.g., LAC diversity and Lastaiders in the workplace; and 3. LACs and compassionate communities including, e.g., the effects on death literacy and grief literacy as well as participation of the public in education via peer education. Future research on PPCE, the LAC, and the LAC-KT should also focus on retention of skills and knowledge over time and long-term effects of the courses.

## Figures and Tables

**Table 1:** Contents of the Last Aid Course.

Module Nr.	Topic	Course Content
Module 1	Dying as a normal part of life	Welcome and introductions First aid and last aid What you can do to care The process of dying
Module 2	Planning ahead	Networks of Support Making decisions Medical and ethical aspects Advance care planning Advance Directive Medical power of attorney
Module 3	Relieving suffering	Typical problems and symptoms Caring/relieving suffering Nutrition at the end of life How to comfort
Module 4	Final goodbyes	Saying goodbye/final farewell rituals Funeral and various forms of burials Grieving is normal Grief and ways of grieving Questions and comments

Duration of the LAC: 3.5 h including four modules with 45 min each plus a break of 30 min.

**Table 2:** Overview of Last Aid studies and reviews.

Articles: Authors, Journal, and Publication Year	Main Topic	Type of Study	Evaluation Method	Informants and Response Rate	Involved Countries	Main Findings
Bollig G, Mainzer K, Fiedler H, Pothmann R. The Last Aid Course for kids and teens from 8–14 years: Preliminary results from the first pilot test. *Hospice & Palliative Medicine International Journal*. 2020 Jan 14;4(1):1–3. [40]	First LAC-KT pilot course	Pilot study	Oral feedback from a group discussion	- Ten kids and teens from 9 to 14 years - 100% participated in an oral feedback round after the course	Germany	LAC-KT is feasible and very well accepted by children and teenagers from 9 to 14 years of age. Children and teenagers want to talk and learn about dying, death, and grief.
Bollig G, Knopf B, Meyer S, Schmidt M. A new way of learning end-of-life care and providing public palliative care education in times of the COVID-19 pandemic: Online Last Aid Courses. *Archives of Health Sciences*. 2020 Sep;4(1):1–2. [41]	Online Last Aid course (OLAC) started during the COVID-19 pandemic	Opinion article	Description of the work to establish an OLAC and proposal for implementation and research	Description of the process of establishing an OLAC by a working group and the very first experiences of the instructors	Germany	Suggestions for implementation of OLAC are provided. The first experiences with both Last Aid courses for citizens and the training of Last Aid Course instructors using online meetings are very encouraging. Scientific evaluation of the OLAC is ongoing.
Bollig G, Meyer S, Knopf B, Schmidt M, Bauer EH. First experiences with online Last Aid Courses for public palliative care education during the COVID-19 pandemic. *Healthcare*. 2021 Feb 5;9(2). 172. [42]	Online Last Aid course (OLAC) started during the COVID-19 pandemic	Prospective mixed methods multicentre study	Questionnaire after course participation and focus group discussions of German Last Aid course instructors	A total of 92 of 156 participants (response rate 59%) from 15 online Last Aid Courses from Germany and Brazil	Germany Brazil	Online Last Aid Courses are feasible and well accepted by the participants. The online format increased the number of participants who are caregivers and younger people. Many participants and instructors prefer classroom teaching, thus the online course was described as the second-best choice only.
Bollig G, Brandt Kristensen F, Wolff DL. Citizens appreciate talking about death and learning end-of-life care: A mixed-methods study on views and experiences of 5469 Last Aid Course participants. *Progress in Palliative Care*. 2021;29(3):140–148. [29]	Evaluation of LAC from Germany, Switzerland and Austria	Prospective mixed-methods multicentre study from three countries	Questionnaire after course participation	A total of 5469 of 6014 Last Aid Course participants from 408 courses in Germany, Switzerland, and Austria (response rate 91%)	Germany Switzerland Austria	The median age of participants was 56 years. A total of 88% were female. A total of 76% of participants rated the course as very good. A total of 99% would recommend the course to others. Last Aid Courses are both feasible and accepted by citizens from different countries and backgrounds. A total of 9.4% of participants had a medical profession.
Mueller E, Bollig G, Becker G, Boehlke C. Lessons learned from introducing Last Aid Courses at a university hospital in Germany. *Healthcare*. 2021 Jul 16;9(7). 906. [43]	LAC for employees of a tertiary university hospital	Prospective study using questionnaires	Pre- and post-survey questionnaire before and after LAC participation	A total of 55 of 56 participants completed an evaluation survey (response rate 98%)	Germany	The most prevalent learning goals were preparation for emotional aspects of care for the dying and for medical/care aspects of care for the dying, and knowledge of supportive services and facilities. Last Aid Courses were more suitable to educate non-medical hospital staff about care for the dying. Medical staff requested courses with an extended curriculum in order to meet their learning goals. Last Aid Courses were well accepted and helped to reduce information deficits on care for the dying.
Bollig G, Safi M, Schmidt M, Ewald H. Is there a need for cultural adaptation of the Last Aid Course? A mixed-methods study across the Danish-German Border. *Healthcare* 2022, *10*(4),658. [44]	LAC for German and Danish participants in the border region	Prospective mixed-methods study	Questionnaire after the LAC and focus group discussions	- A total of 53 of the 79 participants returned a questionnaire (response rate 67%). - A total of 49 of the 79 participants (response rate 62%) joined one of seven focus group discussions in Danish (three focus groups) or German (four focus groups)	Denmark Germany	Almost all participants appreciated the LAC as an option to talk and learn about death and end-of-life care. The informants found individual differences more important than cultural differences in end-of-life care but described differences connected to regulations and organization of services across the border. Suggestions for adaptation and improvement of the LAC included the topics of organization and support across the border, religions, and cultures, and supporting people in grief. The findings of the study will inform a revision of the Last Aid curriculum and future projects across the border and will help to include the views of minorities.
Zelko E, Vrbek L, Koletnik M. Last Aid Course—the Slovenian experience. *Healthcare* 2022, *10*(7), 1154. [45]	Experiences with LAC in Slovenia	Prospective mixed-methods study	Questionnaire after LAC participation	- A total of 386 LAC participants received a questionnaire personally or by e-mail. - A total of 250 questionnaires were returned (response rate 64.7%)	Slovenia	The Slovenian Last Aid experience is comparable to that reported by countries where the course had been previously organized. In Slovenia, the courses were extremely well-received and favourably rated. The adaptation to Slovenian cultural requirements with information from the local communities was supported by materials prepared by the PO-LAST project.
MacAden L, Broadfoot K, Carolan C, Muirhead K, Neylon S, Keen J. Last Aid training online: Participants’ and facilitators’ perceptions from a mixed-methods study in rural Scotland. *Healthcare* 2022, *10*(5), 918. [46]	Evaluation of online Last Aid Courses in Scotland	Prospective mixed-methods study	Combination of online survey of OLAC participants followed by individual semi-structured qualitative interviews with both LAT participants and facilitators	- Of 68 LAC participants, 26 participants completed the survey (response rate of 38%) - Five LAC instructors participated in interviews	Scotland	Provision of foundational death literacy education in social contexts enhances the personal knowledge, skills, and confidence of individual community members and supports the notion that this personal growth could lead to strengthened community action. There is potential to include LAC as the foundational core training to promote death literacy in communities.
Giehl C, Chikradze N, Bollig G, Vollmer HC, Otte I. “I needed to know, no matter what I do, I won’t make it worse”—expectations and experiences of Last Aid Course participants in Germany—A qualitative pilot study. *Healthcare* 2023, *11*(4), 592. [47]	Experiences of LAC participants with caring for seriously ill and dying people	Qualitative pilot study	Semi-structured interviews of LAC participants	Five LAC participants participated in telephone interviews	Germany	Overall, the interviewed participants have a positive attitude toward Last Aid Courses. They perceive the courses as helpful as they provide knowledge, guidance, and recommendations of action for concrete palliative situations. Eight main topics emerged during analysis: expectations regarding the course, transfer of knowledge, reducing fear, the Last Aid Course as a safe space, support from others, empowerment and strengthening of own skills, and the improvement needs of the course. The pilot interviews show initial indications that the impact, as well as supportive and challenging factors regarding the ability to care for relatives to cope, should be explored in further research.
Woodworth J. Building death literacy through Last Aid: An examination of agency, ambivalence and gendered informal caregiving within the Swedish welfare state. *NORA-Nordic Journal of Feminist and Gender Research* 2023. [48]	Evaluation of the implementation of the LAC as an initiative for health-promoting palliative care in Sweden	Case study	Case study with focus on the work of four course instructors in organizing and teaching Last Aid and the experiences of course participants; combination of personalistic observation and verbatim quotation incorporating a mixed-methods approach comprising action research (AR), focus groups, and participant observation	Four course instructors (including the first author) participated in the collection of the qualitative data	Sweden	Participants articulated Last Aid as a tool which helped them reclaim agency amidst the inertia of competing norms and expectations of informal caregiving. The findings indicate that the implementation of Last Aid into the Swedish context is characterized by a tension between norms of institutional caregiving versus community caregiving, representing a case of sociological ambivalence. Women disproportionately perform informal caregiving for seriously ill significant others in an institutional context where state care services are diminishing.
Bollig G, Gräf K, Gruna H, Drexler D, Pothmann R. “We want to talk about death, dying and grief and to learn about end-of-life care”—lessons learned from a multi-center mixed-methods study on Last Aid Courses for kids and teens. *Children*. 2024 Feb 9;11(2):224. [49]	Evaluation of the implementation of LAC-KT and the experiences with the course	Prospective mixed methods study	Questionnaires and focus group interviews	A total of 2534 valid questionnaires were received from 2996 LAC-KT participants aged 7–17 years (response rate 85%). In addition, two focus group interviews of 22 LAC-KT instructors were performed.	Germany	A total of 84% of the participants already had experience with death and dying. A total of 91% found the LAC-KT helpful for everyone. The majority of the participants appreciated the opportunity to talk and learn about death, dying, grief, and palliative care. The LAC-KT is feasible, very well accepted, and a welcome opportunity for exchanging and obtaining information about dying, grief, and palliative care. Further research on the effects and the implementation of the LAC-KT throughout the school curricula and the long-term effects of the training are needed.
Bollig G, Mueller-Koch S, Zelko E. Instructors’ views on and experiences with Last Aid Courses as a means for public palliative care education—A longitudinal mixed-methods study. *Int. J. Environ. Res. Public Health* 2025, 22, 1117. [50]	Instructors’ views on and experiences with Last Aid Courses	Prospective mixed methods study	Questionnaires and focus group interviews	Longitudinal study over 5 years	Germany	The LAC participants felt empowered after the LACs. Continuing development is a characteristic of the LAC project. The positive effects of the LACs included empowerment and positive interactions between the instructors and participants. LACs had a positive impact on all five principles of social space orientation.
Bollig G, Heller A. The Last Aid Course—A simple and effective concept to teach the public about palliative care and to enhance the public discussion about death and dying. *Austin Journal of Palliative Care*. 2016;1(2). [51]	Description of the LAC concept and first experiences with the implementation	Mini review	Review of the literature from a PubMed search and description of the Last Aid concept and the first experiences in practice from three countries	Results from the first German pilot study and general experiences with the course in Germany, Denmark, and Norway are described	Germany Denmark Norway	Public Palliative Care Education is needed. First experiences show that the Last Aid Course is feasible in different countries. The LAC is very well accepted by the participants. The LAC is a simple and effective concept to teach the public about palliative care.
Bollig G, Hjelm Brandt Kristensen F, Ciurlionis M, Knopf B. Last Aid Course. An education for all citizens and an ingredient of compassionate communities. *Healthcare*. 2019 Jan 28;7(1). 19. [52]	Description of the experiences with LAC and review of the literature	Review	Review of the literature on educational efforts regarding palliative care for non-professionals in PubMed/Medline and the existing literature on Last Aid courses	A literature search in PubMed and Medline led to 47 papers with accessible abstracts. Of these 47 papers, only 7 papers included palliative care education for the public as a major topic. Additional sources were found by hand searches in books and reference lists or the Internet.	n.a.	The public requires more information and education about palliative care. The Last Aid Courses are accepted by many people and have an enormous potential to spread information about palliative care throughout the public and can contribute to enhanced public discussion about end-of-life care. More research on the education of non-professionals in palliative care, end-of-life care, and the compassionate community approach is necessary. Research on the implementation of the Last Aid course is ongoing in different countries.
Bollig G, Bauer EH. Last Aid Courses as measure for public palliative care education for adults and children: A narrative review. *Annals of Palliative Medicine*. July 2021;10(7):8242–8253. [13]	A narrative overview of the current knowledge on Last Aid Courses (LACs) and experiences from the implementation process in different countries	Narrative review	A literature search in PubMed/Medline was performed and a narrative overview of the existing literature on LAC and PPCE is provided	A literature search in PubMed using “last aid” led to 6 results only. A search strategy combining “palliative care education” and “public” led to 69 results in PubMed. Fifty-six of these were published within the last 5 years.	n.a.	Measures to raise awareness of palliative care, to improve the public discussion about death and dying, and to empower citizens to participate in end-of-life care in the community are needed and should be offered more widely. LACs are one example of a feasible and well-accepted approach to PPCE in different countries. The LAC can contribute to a public debate on death, dying, and palliative care and may contribute to the empowerment of citizens in the provision of end-of-life care. Further research on PPCE and the LAC is necessary to add to the body of knowledge in this emerging field and is ongoing at present.
Elder AB. A narrative review—characterizing palliative care curriculum aimed at high school adolescents and young adults. *Illness, Crisis & Loss*. Online first 15 April 2025. [53]	Exploration of the characteristics of palliative care curriculum developed for high school adolescent and young adults	Narrative review	Literature search using the databases ERIC, PubMed, and CINHAL	A total of 202 articles were included. These articles were screened using strategic exclusion criteria, resulting in five relevant literature works in the analysis.	n.a.	Studies including a variety of designs, methods, and strategies all resulted in positive participation, feedback, and experiences from high school AYA provided with palliative care curriculum. The literature indicates that the use of subject matter experts within palliative care curriculum for high school students is a core characteristic in meeting the World Health Organizations recommendation to embed palliative care curriculum into public awareness strategies.
Seckin M, Tiwana R, Fry D, Bailey C. Key themes and approaches in palliative and end-of-life care education for the general public: A systematic review. *BMC Palliative Care* 2025 24:219. [54]	Identification of key educational components related to palliative and end-of-life care for citizens, volunteers, and the general public	Mixed-method systematic review	A mixed-methods systematic review was conducted, incorporating four electronic databases (MEDLINE, PsycINFO, CINAHL, and the Cochrane Library) and grey literature searches, and quality was assessed using Hawker et al.’s (2002) critical appraisal checklists	A total of 20 studies with 10.037 participants were included in the systematic review	n.a.	The analysis revealed six main themes: foundational concepts and philosophies, communication and decision-making, planning and preparation, symptom management, end-of-life care practices, and caregiving support. The review highlights the importance of training programmes to improve community involvement in caregiving and enhance the quality of care for individuals with life-limiting conditions. Expanding access to such educational resources can empower more people to contribute confidently to end-of-life care in their communities. The LAC is one the concepts that includes all six main themes (out of 16 different educational programs).

LAC = Last Aid Course (for adults), OLAC = online Last Aid Course (for adults), LAC-KT = Last Aid Course for kids and teens, PPCE = Public Palliative Care Education, and n.a. = not applicable.

## Data Availability

No new data were created or analyzed in this study. Data sharing is not applicable to this article.

## Abbreviations

The following abbreviations are used in this manuscript:

gGmbH	gemeinnützige Gesellschaft mit beschränkter Haftung (non-profit non-governmental organization in Germany)
LAC	Last Aid Course
LAC-KT	Last Aid Course Kids and Teens
LARGI	Last Aid Research Group International
OLAC	Online Last Aid Course
NGO	Non-governmental Organization
PHPC	Public Health Palliative Care
PPCE	Public Palliative Care Education
n.a.	Not Applicable
